# Study of the Polycarbonate-Urethane/Metal Contact in Different Positions during Gait Cycle

**DOI:** 10.1155/2014/548968

**Published:** 2014-08-27

**Authors:** Sergio Gabarre, Antonio Herrera, Jesús Mateo, Elena Ibarz, Antonio Lobo-Escolar, Luis Gracia

**Affiliations:** ^1^Department of Mechanical Engineering, Engineering and Architecture School, University of Zaragoza, María de Luna 3, 50018 Zaragoza, Spain; ^2^Department of Surgery, Medicine School, University of Zaragoza, Domingo Miral s/n, 50009 Zaragoza, Spain; ^3^Aragón Health Sciences Institute, Avenida San Juan Bosco 13, 50009 Zaragoza, Spain; ^4^Department of Orthopaedic Surgery and Traumatology, Miguel Servet University Hospital, Avenida Isabel la Católica 3, 50009 Zaragoza, Spain

## Abstract

Nowadays, a growing number of young and more active patients receive hip replacement. More strenuous activities in such patients involve higher friction and wear rates, with friction on the bearing surface being crucial to ensure arthroplasty survival in the long term. Over the last years, the polycarbonate-urethane has offered a feasible alternative to conventional bearings. A finite element model of a healthy hip joint was developed and adjusted to three gait phases (heel strike, mid-stance, and toe-off), serving as a benchmark for the assessment of the results of joint replacement model. Three equivalent models were made with the polycarbonate-urethane Tribofit system implanted, one for each of the three gait phases, after reproducing a virtual surgery over the respective healthy models. Standard body-weight loads were considered: 230% body-weight toe-off, 275% body-weight mid-stance, and 350% body-weight heel strike. Contact pressures were obtained for the different models. When comparing the results corresponding to the healthy model to polycarbonate-urethane joint, contact areas are similar and so contact pressures are within a narrower value range. In conclusion, polycarbonate-urethane characteristics are similar to those of the joint cartilage. So, it is a favorable alternative to traditional bearing surfaces in total hip arthroplasty, especially in young patients.

## 1. Introduction

The modern age of total hip arthroplasty (THA) began with Charnley in the 60s of past century [1]. This technique has entailed one of the greatest advances in orthopaedic surgery. Since long term survival of implants is a surgeon's primary goal, over the past half-century there have been important developments in implant designing, implant to bone fixation techniques, and bearing surfaces [2].

Nowadays, a growing number of young and more active patients receive hip replacement [3]. Increased and more strenuous activities in such patients involve higher friction moments and higher wear rates [4, 5]. As activity increases friction on the bearing surface increases accordingly, leading to a temperature rise which is a risk factor for implant stability in the long term [6]. As a consequence, increased friction can contribute to an aseptic loosening of implants in the mid and long term [7, 8]. Thus, friction reduction becomes crucial for the long term survival of hip arthroplasty [6].

The use of bearing surfaces with low wear rates is essential to prevent wear debris, which could trigger immunological reactions leading to aseptic loosening. At present, the most common bearing surfaces are as follows.

### 1.1. Metal on Metal Bearing (M-o-M)

Apart from some historical precedents, McKee and Watson-Farrar designed in the 60s the first hip prosthesis with a M-o-M bearing surface [9]. Since then, several alternatives have emerged until hip resurfacing appeared in the 90s [10]. Although M-o-M bearings have a low friction coefficient (*μ* = 0.004), low wear rate (3.5 mm/year), and smaller wear debris particles than metal on polyethylene (UHMWPE) bearing couple [11], the risk of adverse biologic reactions caused by metallic debris and pseudotumors [12–15] has brought into question M-o-M bearings. Nevertheless, classic M-o-M hip implants, with mild contents of cobalt and chromium, have achieved superb long term outcomes [16–19]. We agree with Migaud et al. [20] that M-o-M bearing couplings are still useful provided that surgical indication, implant design, and surgical technique are suitable.

### 1.2. PE on Metal Bearing (PE-M)

Polyethylene-metal bearing was introduced by Charnley more than fifty years ago. Its friction coefficient is within a range of 0.05–0.15. Conventional polyethylene has a high wear rate. The biological response to wear debris particles can lead to aseptic loosening of the femoral or, more often, the acetabular component. About 20% to 40% of all revision hip arthroplasties are due to aseptic loosening of implants [21]. Highly cross-linked polyethylene has lowered the amount of wear by 50–62% according to various studies [22–27]. But its use is discouraged in young patients with a high activity and long life expectancy [6–8].

### 1.3. Ceramic-on-Ceramic (C-o-C)

Ceramic bearing surfaces were first introduced by Boutin in the 70s of past century [28] and have experienced a significant evolution. The introduction of the hot isostatic pressing (HIP), during the 90s, increased alumina's durability and the emergence of alumina matrix composite, at the beginning of the 21th century, improved mechanical wear resistance [29]. This fourth generation of composite ceramics of alumina matrix (BIOLOX Delta, CeramTec GmbH, Germany) is composed of 82% of alumina and 17% zirconia. Improved oxidation resistance, hardness and wear were achieved adding a 0.5% of chromium oxide [30]. C-o-C bearing has a friction coefficient of 0.09 and exhibits minimal wear (3.9 *μ*m/year is estimated [31]). Some authors state that ceramic debris particles are within the same size range as those from polyethylene (mean size of 0.7 microns), which are able to generate a biologic response [32]. Conversely, a comparative work in the literature reported smaller size for wear particles of C-o-C (10–30 nm) than polyethylene particles (0.1–1.0 micron) [33]. Its major limitations are the dramatic cracking failure consequences and squeaking. Ceramic bearing surfaces are considered to be an excellent choice in THA in young patients [27, 30, 34, 35].

### 1.4. Polyethylene-Ceramic Bearing

The combination of polyethylene acetabular liners with zirconia femoral heads [36] began to be used in the 1980s. It has been proven that, after walking for an hour, a significant rise in temperature is produced inside the joint [37], which could contribute to polyethylene wear [38, 39]. In accordance with some authors this bearing coupling does not offer advantages over the polyethylene on metal option [40]. Nevertheless, there is some controversy about this aspect as recent works reported in the literature outlined that the lowest wear rate of PE is achieved when combined with ceramic Biolox heads [41–43].

### 1.5. Polycarbonate-Urethane-Metal Bearing (PCU-M)

Friction minimizing between bearing surfaces remains crucial to ensure THA survival in the long term [6]. At the outset, clinical use of polyurethane was hampered by its manufacturing processes [44]. Over the last years, the polycarbonate-urethane Bionate 80A production has offered a feasible alternative to conventional bearings. Its biostability and high resistance to hydrolysis, oxidation, and calcification have been demonstrated in vitro. [45]. Three-year follow-up, in vivo studies have proved the absence of appreciable signs of biodegradation [46]. Comparative wear studies between PCU on metal and highly cross-linked polyethylene (including gamma irradiation) on metal over several million loading cycles showed that polyurethane has a lower wear rate, better corrosion resistance, and wear debris particles less prone to cause osteolysis [47–51]. All these polyurethane features make it a favorable alternative as THA bearing surface.

There is growing interest in this new coupling which has been subject of study in recent years. To our knowledge, no finite elements simulation study on this kind of material has been conducted so far. Therefore, our study can be assumed as an innovative research which supports and is added to all the aforementioned related works.

The aim of this work is to analyze the contact pressures generated during gait cycle in the polycarbonate-urethane/metal bearing, comparing the results with the corresponding to a healthy joint. Several finite element (FE) models were implemented in order to simulate different situations, both in the healthy joint and after total hip replacement. Three stages of the gait cycle were studied: heel strike, mid-stance and the late stance peak toe off.

## 2. Materials and Methods

A first FE model of a healthy hip joint was developed. The geometry of the model was obtained from a femur, pelvis, and sacrum of a 65-year-old male donor. A computed tomography (CT) scan (512 × 512 acquisition matrix, FOV = 240 mm, slice thickness = 0.5 mm in plane resolution) was obtained using a TOSHIBA Aquilion 64 scanner (Toshiba Medical Systems, Zoetermeer, The Netherlands). Stacks of images from each bone are processed using Mimics Software (Materialise, Leuven) [52]. Polylines referred to cortical and trabecular bone are exported to I-deas 11 NX Series software (Siemens, Plano, Texas) [53].

Afterwards, each of the bone components of the hip joint was examined by means of a 3D Roland PICZA (Irvine, California) scanner in order to get a better precision of the outer geometry. This device has a 0.2 mm voxel resolution and two sweep modes, rotational and plane-based type. The scanner provides a cloud of points representing the initial geometry and, by means of its own software, initial cleaning operations are performed: deleting abnormal surfaces and pulling the 3D image out of the scan noise. The next step is to convert the latter geometry to a polygonal mesh with the Roland Pixform software (Irvine, California) [54]. In contrast to the first one, this software allows us a deeper geometry processing: global and local operations can be made by registering different scans of the same bone in order to get a wrapping group of surfaces where total number and order of their points of control can be modified.

The final geometry is imported to I-deas 11 NX Series software [53], where it is combined with the inner geometry previously obtained via CT scans. Based on the external and internal geometries, transition from cortical to cancellous bone is determined.

After defining the geometry of the different materials, the mesh can be generated. A sensitivity analysis was performed to determine the minimal size mesh required for an accurate simulation of contact. For this purpose, a mesh refinement was performed in order to achieve a convergence towards a minimum of the potential energy, both for the whole model and for each of its components, with a tolerance of 1% between consecutive meshes.

Due to the difficulties in obtaining accurate soft tissue images in CT scans, a methodology was developed to generate, into the model, those soft tissues needed to keep the contact between bearing surfaces. Geometry of the acetabular socket and the femoral head was used as a baseline to build up the geometry of both cartilages, which also come into contact with the labrum. According to several anatomical 3D atlas, three different zones of thickness were sketched onto the bone geometry, ranging from 0.5 mm to 2.0 mm. Auxiliary splines were used to shape several sections, from which both cartilage volumes were created. A similar technique was used to generate the labrum around the acetabular rim. A structured mesh of hexahedral elements was generated in both cartilages. This type of elements is more suitable for solving contact problems than tetrahedral ones [55].

**Figure 1:**
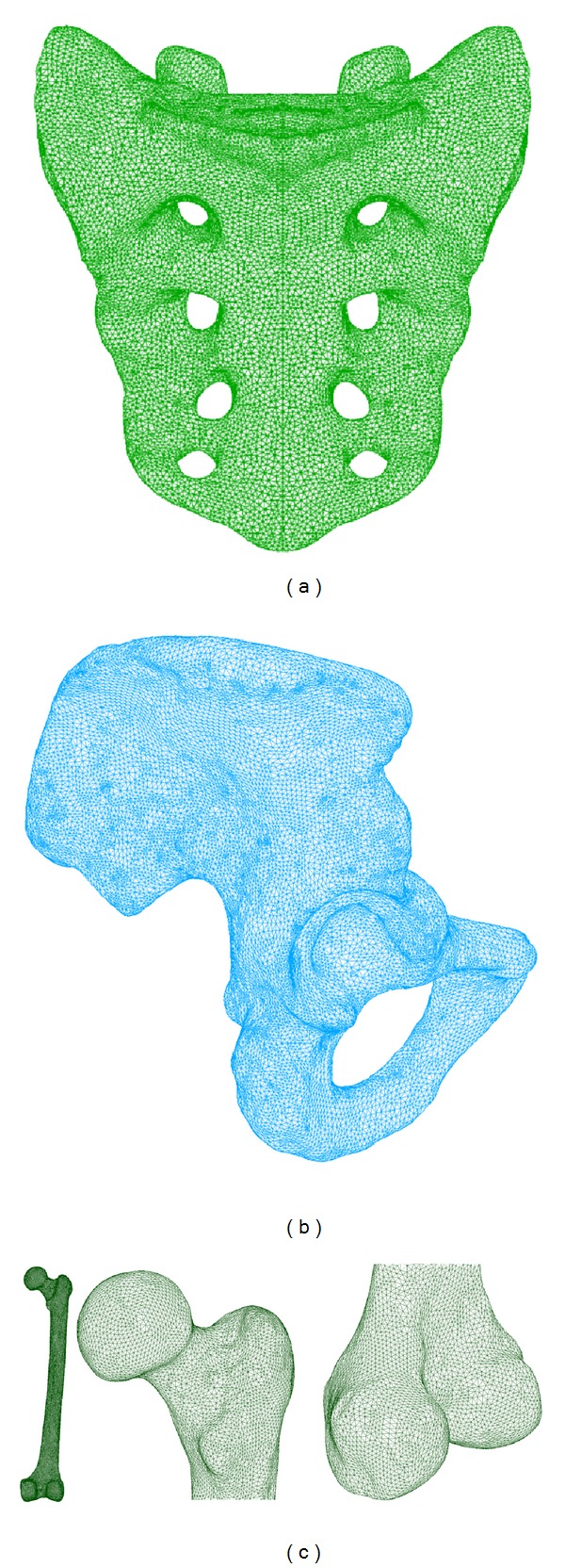
Final FE model of bones: (a) sacrum; (b) pelvis; (c) femur.

**Figure 2:**
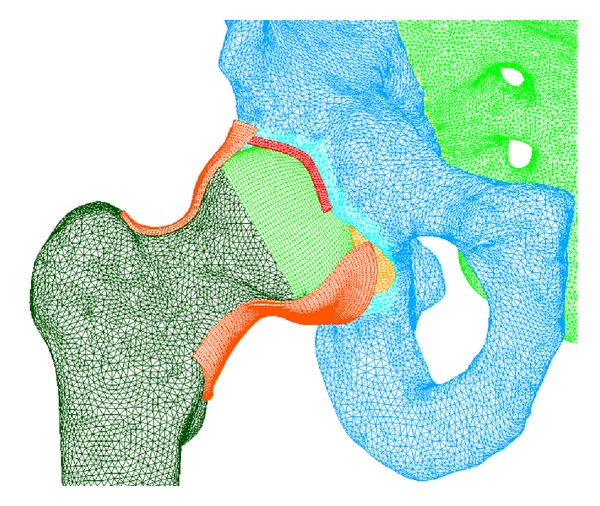
Final model of the complete healthy hip (joint's capsule is cut for a better visibility).

After being meshed, the set of soft tissues and bones are joined in a unique model: the complete model of the hip joint. The final model is shown in Figures 1 and 2. The statistics of the FE model are presented in Tables 1 and 2.

Three FE models were made, one for each of the three most representative phases of gait cycle: heel strike, mid-stance and toe off. These three gait situations are simulated matching the behaviour of healthy model with the Tribofit system model [56]. The healthy joint model serves as a benchmark in the assessment of the results of joint replacement model. In the arthroplasty model, the Tribofit system buffer with shell was used. The system consists of a metallic shell which is inserted directly on the acetabulum and a PCU buffer insert placed on the inside of the shell (buffer-with-shell configuration). The femoral component consists of a spherical metallic head (Cobalt-Nickel-Chromium-Molybdenum alloy), a femoral stem (Ti6-Al4-V alloy), and a stem sleeve which is fixed on the neck of the stem to match the cone of the femoral head.

All the prosthetic components, with the exception of the femoral stem, can be modelled from analytical geometries. The geometry of the stem is more complex; therefore, the same procedure followed for the bones is applied to the stem. After obtaining the geometry of the implant, a surgery was done at the Department of Orthopaedic and Trauma Surgery of the Miguel Servet University Hospital. A senior surgeon implanted the femoral stem and the Tribofit buffer and shell in the cadaveric bones used in our study.

**Figure 3:**
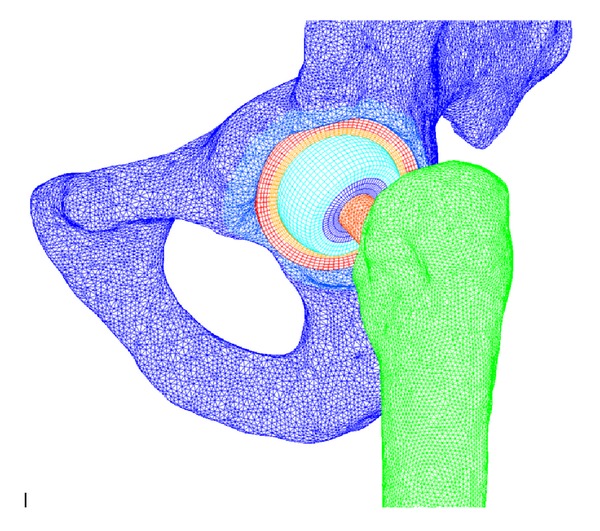
FE complete model with hip implant.

Afterwards, a new 3D scan of the entire ensemble was made, and the surgical procedure was reproduced with the I-DEAS software. In this way, we ensure that implant's alignment and bone cuts are similar to those achieved in surgical conditions. The final FE model is shown in Figure 3. To guarantee the accuracy of the FE results, a sensitivity analysis was performed with a mesh refinement in order to achieve a convergence towards a minimum of the potential energy, with a 1% tolerance between consecutive meshes, for both the healthy and implanted models. The statistics of the FE model are presented in Tables 3 and 4. Material properties are included in Table 5 [57, 58].

**Figure 4:**
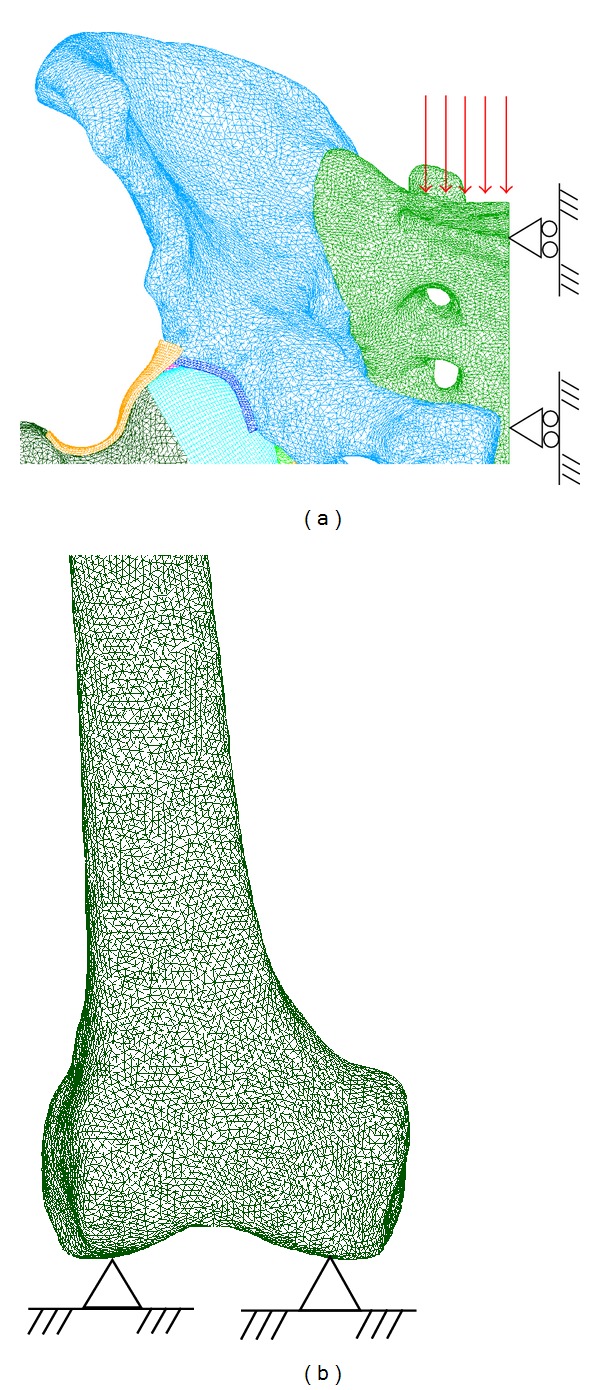
Boundary conditions: (a) load and restraints on sacrum; (b) restraints at femoral condyles.

Three gait phases were simulated: heel strike, midstance, and toe-off, developing three different FE models. The pelvic and the sacral bones were kept in same position in the three models, whereas femur was positioned 10° in anteversion (heel strike) and 0° (mid-stance) and 15° in retroversion (toe-off), respectively (according to bony landmarks described in [59]). Although the whole pelvis model was developed, only a hemipelvis model has been calculated and postprocessed for each group of models: the right side of the pelvis is defined as the healthy model and the left side is modeled as the operated one. Sacrum's sagittal plane is defined as the boundary limit. In both cases load is vertically applied at the top of the sacrum, and femur is fully constrained at its condyles, as shown in Figure 4. Body-weight (BW) loads where considered according to orthoload's database [59]: 230% BW toe-off, 275% BW mid-stance and 350% BW heel strike.

In addition, another key point to be studied is the comparative biomechanical function in different situations. This requires standardising the conditions of loads analysis and path contact distances. Computation and postprocessing were done using Abaqus version 6.12 program (Dassault Systèmes, Providence, Rhode Island) [55].

## 3. Results

Results of the healthy model are presented for the three analyzed phases of gait, showing contact pressures between the femoral and the acetabular cartilages. Similarly, contact pressures between the buffer and the metallic femoral head are shown in the prosthetic Tribofit model. Healthy model acts as benchmark for comparison of the prosthetic model results. Since contact tracks are tridimensional and, at times, quite irregular, two representative and nearly perpendicular paths traversing the maximum are chosen. Both paths provide information in the most relevant directions.

**Figure 5:**
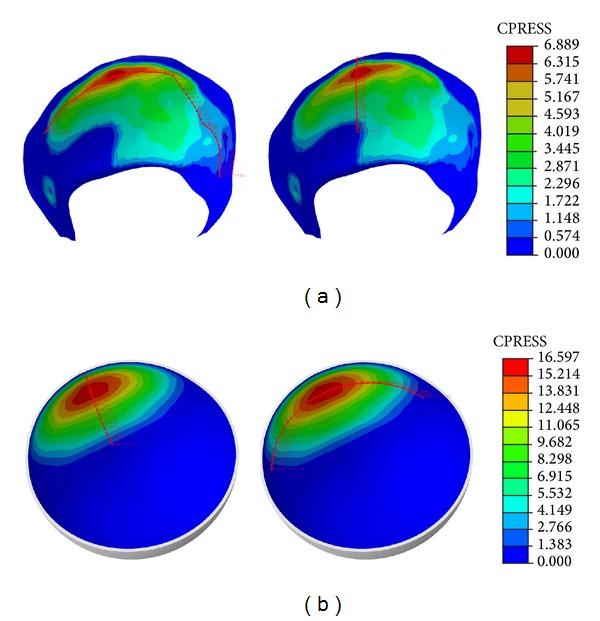
Contact track in midstance: (a) healthy model; (b) model with PCU.

Results corresponding to the midstance phase are shown in Figure 5. So, Figure 5(a) illustrates the contact pressure map in the acetabular cartilage for the healthy joint, including the paths used for procesing the values shown in Figure 5, whereas Figure 5(b) corresponds to the contact pressure map for the polycarbonate-urethane cup in the replaced hip joint, including again the processed paths.

**Figure 6:**
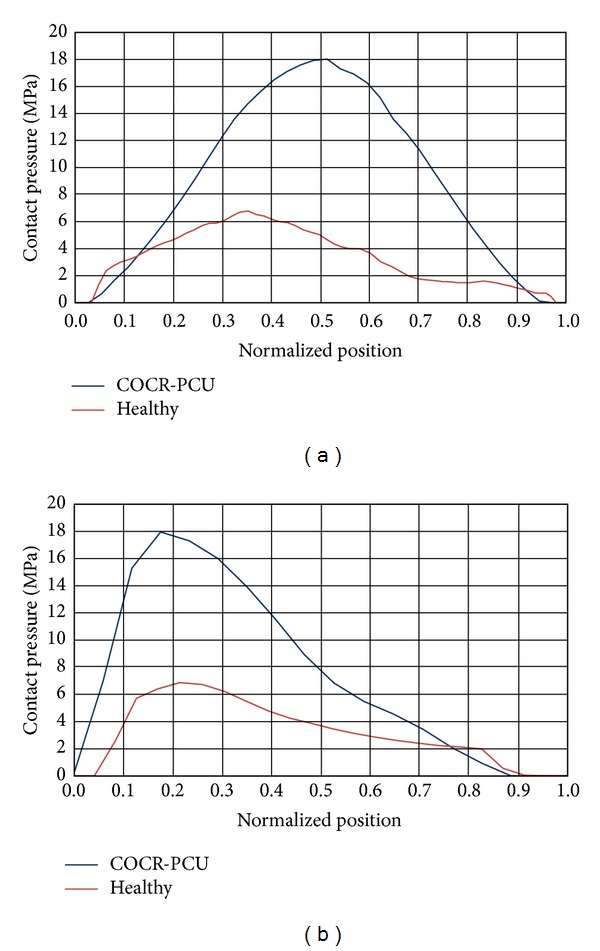
Comparative charts in mid-stance: (a) longitudinal path; (b) transversal path.

As can be seen in Figure 5, contact surface is smaller in the polycarbonate-urethane cup than in the acetabular cartilage and, consequently, the maximum contact pressure peak is higher in the former (Figure 6). The peak ratio between the two models is 2.57.

**Figure 7:**
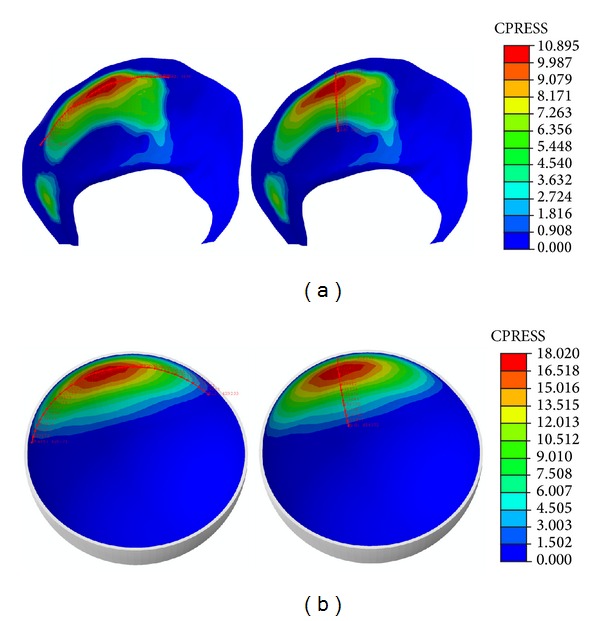
Contact track in heel strike: (a) healthy model; (b) model with PCU.

**Figure 8:**
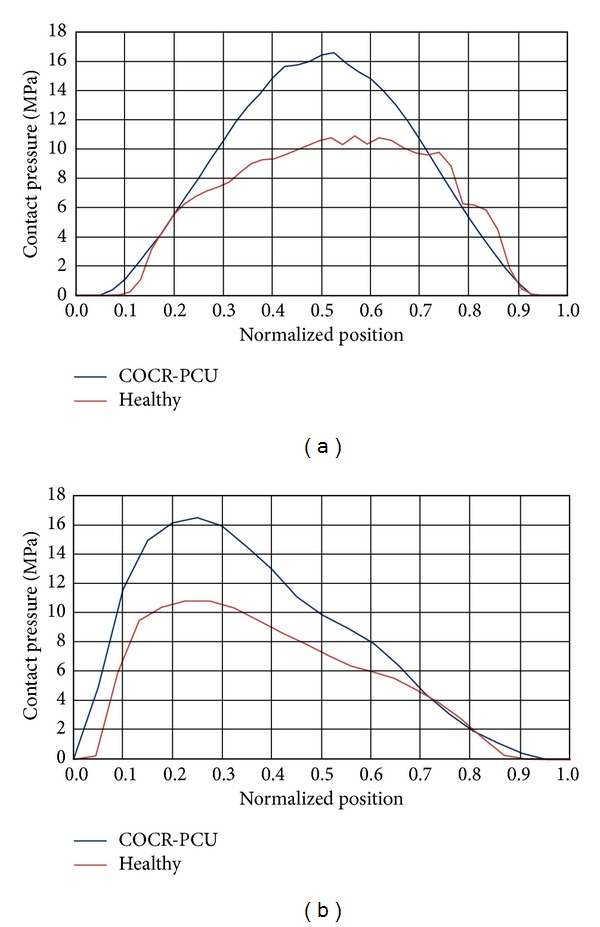
Comparative charts in heel strike: (a) longitudinal path; (b) transversal path.

In the same way, heel strike results are presented in Figure 7.  Figure 7(a), shows a bicentric contact pattern more marked than in the mid-stance phase, which is congruent with the femur positioning relative to the acetabulum. When compared to PCU joint, contact areas are similar and so, contact pressures are within a narrower value range (Figure 8). The peak ratio between the two models is 1.6.

**Figure 9:**
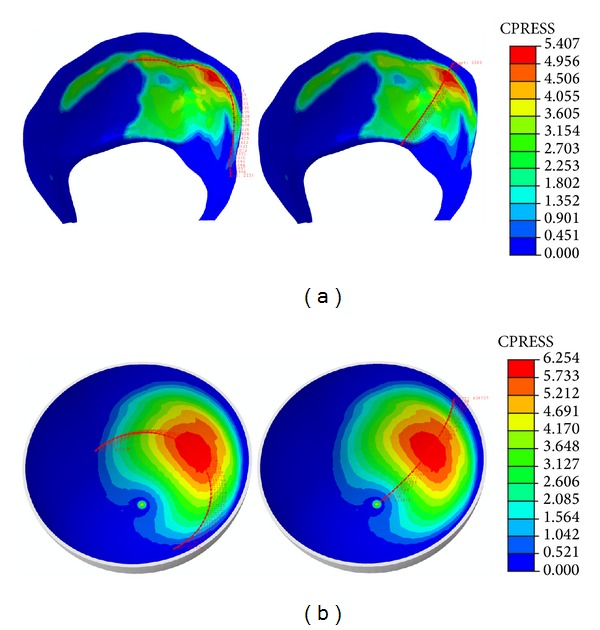
Contact track in toe-off: (a) healthy model; (b) model with PCU.

**Figure 10:**
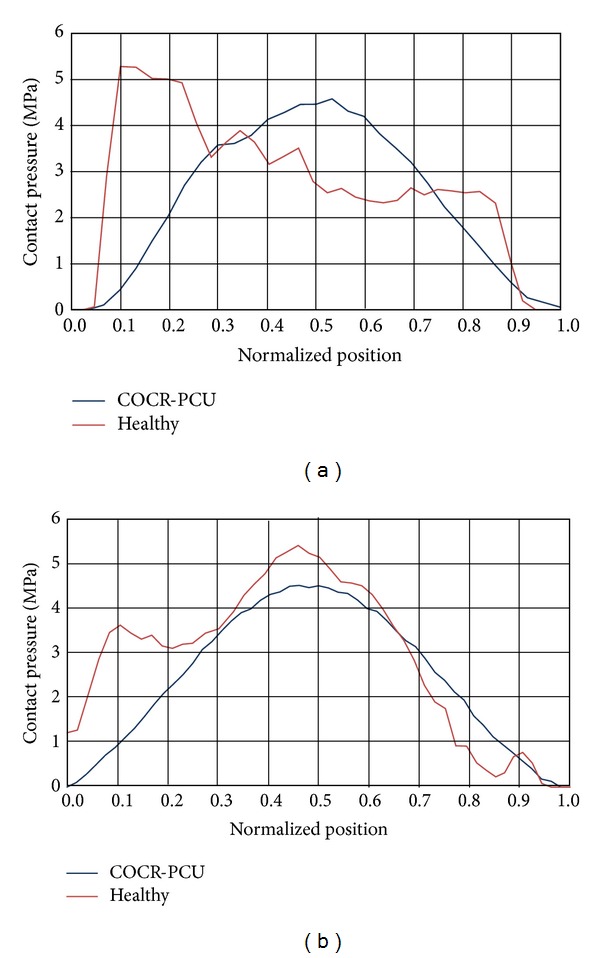
Comparative charts in toe-off: (a) longitudinal path; (b) transversal path.

Finally, it can be observed as the contact area migrates forward in the toe-off phase. Although the contact pattern of the healthy model is bicentric, it is less pronounced than in the midstance phase (see Figure 9). Contact areas in the healthy and operated models are similar; thereby, contact pressure ratio is nearly 1 (see Figure 10).

In view of this result, it can be concluded that PCU is a new bearing surface with stiffness close to that of healthy cartilage. That is why contact areas and pressures are in the same range of the healthy hip, mimicking the healthy cartilage accurately.

## 4. Discussion

In this paper, a pioneer investigation based on finite element simulations of hip arthroplasty with the new PCU soft bearing surface is presented.

Two finite element models of the entire hip joint were developed. The first one reproduces in a very reliable way the bone structure of the human hip joint, including cartilages and soft tissues, in order to assess the joint contact pressures during different phases of gait cycle. The second model was developed after replacing the hip joint with a metallic acetabular shell with a PCU buffer insert and a femoral stem with a metallic head, using a regular surgical technique. All the components were implanted at the donor's bones which had been used to develop the healthy model, so that the models were comparable. Simulations for the three gait phases were conducted in both models. Joint contact pressures were calculated in both models and were compared against each other.

The healthy model exhibits a wide contact pattern. Contact pressures reached 6 MPa for midstance, 10 MPa for heel strike, and 5 MPa for toe-off phase. These values are in range of other previous studies which stands the peak range in 8–12 MPa [57, 60].

The operated model with PCU buffer showed a narrower, but more uniform, contact pattern than the healthy model as a result of better geometry accuracy. Peak contact pressures reached 16.6 MPa in the midstance phase, 18 MPa in the heel strike, and 4.6 MPa in the toe-off phase. In the toe-off phase the whole “contact dome” is developed, producing a peak contact pressure even slightly lower than that of the healthy model. All the aforementioned values are very close to those obtained in healthy hip model simulations. However, the latter are much lower than values obtained with other bearing surfaces: ceramic-on-ceramic 40–250 MPa [61], metal-on-metal 200 MPa [62], metal-on-ceramic 40–112 MPa [63], and polyethylene-on-metal 22 MPa [63]. Contact pressure values are important because they modify the tribologic behavior of the implant and the wear rate over the time. Clearly, contact pressure values in the PCU model closely approximate to those of healthy model. Moreover, hydrophilic PCU feature promotes lubrication between bearing surfaces reducing wear. This fluid lubrication film is similar to that in the healthy joint and is thicker than in other bearing surfaces, providing an excellent lubrication [64]. Low contact pressure values combined with an improved lubrication result in a low wear debris rate [65].

Biocompatibility, biostability, and high oxidation, hydrolysis, and calcification resistance of PCU have been proved [45], and in vivo studies have confirmed its high biodegradation resistance [46]. All these features, together with a lower wear debris rate as compared with highly cross-linked polyethylene, good corrosion resistance, and osteolysis absence [47–51], make PCU a very suitable material for joint implants fabrication. Some previous studies have analyzed retrieved PUC buffers, measuring a minimum volumetric wear rate (1.4–14 mm^3^/year), and a low number of wear debris particles without tissular reaction [66, 67], supporting previous in vivo studies [46]. Available clinical studies are based on small samples with a limited follow-up [68]. Long term follow-up studies are needed to ascertain that PCU can be an alternative to traditional bearing surfaces.

Two options have been suggested for using the PCU buffer. The first one uses PCU as a bearing surface interposed between the metal shell and the spherical metal head. Mechanically, this is the most suitable way of use in our opinion, because metallic wear debris and its harmful impact are prevented [69], improving the long term outcome of the implant without the issues of metal on metal bearing surface.

Another possibility is to fit the PCU buffer directly onto the osseous joint surface of the acetabulum. In this case, PCU might be used as a total hip replacement in osteoarthritis, or as a hip replacement in femoral neck fractures. Though our experience in femoral neck fractures has been satisfactory in the short term, a recent paper presents bad results in fracture cases [70]. In elderly patients with osteoporotic bone, implant selection must be balanced with an individualized patient assessment. It can be difficult to get an adequate press fit of the buffer when bone quality is too poor. In such cases, this way of use of the “buffer-on-bone” is contraindicated

A limitation of the study is the lack of comparison with experimental testing, due to the absence of specific references in the specialized literature about PCU contact stresses. However, as is usual for a correct validation of FE models, a sensitivity analysis was performed to determine the minimal size mesh required for an accurate simulation of contact. The final mesh was achieved after a mesh refinement performed considering convergence towards a minimum of the potential energy, with a tolerance of 1% between consecutive meshes.

## 5. Conclusion

As conclusion from the obtained results, PCU biomechanical characteristics are similar to those of the joint cartilage so it is a favorable alternative to traditional bearing surfaces in total hip arthroplasty, with lower and closer to physiological contact pressures values, especially in young patients. Although clinical experience is still limited PCU could prevent the complications caused by wear debris, such as osteolytic lesions and aseptic loosening, improving the long term survival of hip implants.

## Figures and Tables

**Table 1:** Mesh statistics of the healthy model: bone.

Healthy model	Cortical bone	Cancellous bone	Element type
Femur	164818	63689	4-node linear tetrahedron
Pelvis	176729	39456	4-node linear tetrahedron
Half sacrum	92055	34505	4-node linear tetrahedron

**Table 2:** Mesh statistics of the healthy model: soft tissues.

Healthy model	Number of elements	Element type
Acetabular cartilage	12420	8-node linear brick
Femoral cartilage	12384	8-node linear brick
Labrum	6203	4-node linear tetrahedron
Capsule	36288	8-node linear brick
Fovea	7345	4-node linear tetrahedron
Transverse ligament	2328	4-node linear tetrahedron

**Table 3:** Mesh statistics of model with Tribofit: bone.

Model with Tribofit	Cortical bone	Cancellous bone	Element type
Femur	93708	300036	4-node linear tetrahedron
Pelvis	337081	401196	4-node linear tetrahedron

**Table 4:** Mesh statistics of model with Tribofit: prosthesis.

Model with Tribofit	Number of elements	Element type
Shell	11808	6-node linear triangular prism, 8-node linear brick
Buffer	12672	6-node linear triangular prism, 8-node linear brick
Stem	31162	4-node linear tetrahedron
Mini stem	22773	4-node linear tetrahedron
Spherical head	7344	6-node linear triangular prism, 8-node linear brick
Stem sleeve	4608	6-node linear triangular prism, 8-node linear brick

**Table 5:** Material properties.

Elastic isotropic	Young modulus [MPa]	Poisson ratio
Cortical bone [58]	20000	0.3
Trabecular bone [58]	959	0.12
Cancellous bone [58]	1	0.3
Implant∗	214000	0.3
Stem∗	110316	0.3

Hyperelastic Mooney-Rivlin	*C*_10_ [MPa]	*C*_01_ [MPa]

Buffer∗	2.912	−1.025

Hyperelastic Neo-Hookean	*G* [MPa]	*K* [Mpa]

Cartilage [57]	13.6	1.359

∗Values supplied by the manufacturer [56].

## Abbreviations

BW: Body weight
THA: Total hip arthroplasty
FE: Finite elements
PCU: Polycarbonate-urethane
UHMWPE: Ultra high molecular weight polyethylene
CT: Computed tomography
3D: Three-dimensional.
